# Prevalence of post partum modern family planning utilization and associated factors among postpartum mothers in Debre Tabor town, North West Ethiopia, 2018

**DOI:** 10.1186/s13104-019-4464-0

**Published:** 2019-07-17

**Authors:** Eden Bishaw Taye, Dawit Gebeyehu Mekonen, Tibeb Zena Debele

**Affiliations:** Department of Midwifery, College of Medicine and Health Science, University of Gondar, P.O. Box 196, Gondar, Ethiopia

**Keywords:** Postpartum family planning, Cross-sectional study, Debre Tabor town

## Abstract

**Objective:**

The aim of this study was to assess the prevalence of postpartum modern family planning utilization and associated factors among postpartum mothers in Debre Tabor town, North West Ethiopia, 2018.

**Result:**

The proportion of postpartum modern contraceptive utilization in Debre Tabor town was 63% (95% CI 59%, 67.4%). In multivariable logistic regression analysis; age of the mother (25–29) [AOR: 2.004 (95% CI 1.079, 3.722)], married women [AOR 4.804 (95% CI 1.843, 12.521)], return of menses [AOR: 3.639 (95% CI 2.454, 5.396)] and previous history of family planning [AOR: 2.409 (95% CI 1.474, 3.937)] were the factors positively associated with utilization of postpartum modern contraceptive.

## Introduction

Postpartum family planning (PPFP) is the prevention of unintended and closely spaced pregnancies through the first 12 months following childbirth [1]. Thus, after delivery mothers are counseled and encouraged to initiate modern contraceptive method with in the specified period.

If a women had only the number of pregnancies they wanted, at the intervals they wanted, maternal mortality would drop by 30% [2]. Family planning (FP) can avert 3.2 million out of 5.6 million under five deaths and 109,000 out of 155,000 (70%) of maternal deaths. However, A Demographic health survey data from 57 countries indicated that, right after delivery 62%, after 6 months of amenorrhea 43% and at the end of amenorrhea 32% of women in the first year after birth have an unmet need for contraception [3].

If a woman is amenorrhic, fully or nearly fully breast feeding and in the first 6 months of delivery, the risks of getting pregnant will be reduced by 98%. Therefore, post-partum mothers have a special privilege of using lactational amenorrhea (LAM) as one of the alternatives of family planning methods [1–3]. The 6th month period however, is a hallmark of shifting from exclusive breastfeeding to complementary feeding [1]. It is also a period where LAM users should shift to other modern contraceptives methods.

Pregnancies that occur in the first year of the delivery are mostly unplanned and risky for the mothers. It also results in adverse birth outcomes for the babies such us, preterm, low birth weight, and small for gestational age [2, 4]. If couples spaced their pregnancies at least 2 years apart from the previous birth, the morbidity and mortality risk both for the mothers and their babies will be reduced. In addition, spacing births allows parents to devote more time to each child in the early years [5, 6].

Although the majority of postpartum women indicated the desire to delay the next birth; mostly, either family planning methods are not offered to, or taken up by the women adequately after delivery or in the first year of postpartum [7].

Therefore, this research tries to assess the prevalence and factors associated with the PPFP utilization there by help policy makers create a strategy to tackle the problem and increase the CPR as high as possible and improve the quality of life both for the mother and her new born baby.

## Main text

### Methods

#### Study design and setting

A community-based cross-sectional study was conducted from September 20 to October 20, 2018, at Debre Tabor town which is the capital city of South Gondar zone Amhara regional state, North West Ethiopia. Based on the 2007 National Census conducted by Central Statistical Agency of Ethiopia (CSAE) projection, the town has a total population of 55,596 of whom 27,644 (49.7%) were male and 27,952 (50.3%) were female. From the total population, 96.72% are Orthodox Christian, and 2.54% are Muslim [8].

#### Sample size and sampling procedure

Post-partum women between 6 and 12 months of delivery who are permanent residents of Debre Tabor town were included in the study. A single population proportion formula was used to calculate the sample size of 550. The following assumptions were made to calculate the sample size, magnitude of PPFP utilization 68.1% [9] from a previous study, 95% confidence interval, 5% margin of error, a design effect of 1.5 and nonresponse rate of 10%. Three out of the six Kebeles (kebele1, kebele2, and kebele5) were selected by simple random sampling Then, the study participants were all mothers between 6 and 12 months of delivery in the selected Kebeles.

#### Operational definitions

*Postpartum period:* The period between 6 and 12 months of delivery [9].

*Utilization of PPFP*: If a woman is using any one of the following modern contraceptive methods between 6 and 12 months following her recent childbirth: pill, intrauterine device, injectable, condom (men or women), women sterilization, implants [9, 10].

#### Data collection instrument and process

Data were collected by face-to-face interview using a structured and pre-tested questionnaire. The questionnaire was prepared in English, translated to Amharic and translated back to English. The data collection was performed by six diploma midwives and supervised by two MSc midwives.

#### Data analysis

Data were entered with epi-info 7 and analyzed using SPSS version 20. Bivariate and multivariable logistic regression analyses were conducted to identify predictors for PPFP utilization. Variables found to be significant on Bivariate analysis (P < 0.2) were included in the multivariable model. Adjusted odds ratios and 95% confidence intervals were generated. Probability values < 0.05 were considered statistically significant.

### Results

#### Socio-demographic characteristics

From a total of 563 postpartum mothers in the selected Kebeles, 546 mothers were included in the study, making a response rate of 97%. The mean age of the respondent’s was 27.57 years with SD of 4.781. From the participants, 519 (95.1%) were married (Table 1).

**Table 1:** Socio-demographic characteristics of postpartum mother at Debre Tabor town, south Gondar zone, North West Ethiopia, 2018 (n = 546)

Variables	Category	Frequency	Percent
Age	≤ 24	130	23.8
	25–29	246	45.1
	30–34	105	19.2
	≥ 35	65	11.9
Marital status	Married	519	95.1
	Others^a^	27	4.9
Religion	Orthodox	529	96.9
	Muslim	15	2.7
	Others^b^	2	0.4
Level of education	No formal education	113	20.7
	Primary	92	16.8
	Secondary	141	25.8
	Tertiary	200	36.6
Occupation	Housewife	331	60.6
	Government and private employed	112	20.5
	Laborer	6	1.1
	Self-employed	61	11.2
	Others^c^	36	6.6
Partners educational status (N = 532)	No formal education	101	19.0
	Primary education	71	13.3
	Secondary education	131	24.6
	Tertiary education	229	43
Partners occupation (N = 532)	Gov’t employed and private employed	261	49.1
	Laborer	59	11.1
	Self-employed	186	35
	Others^c^	26	4.9

^a^Others include single, widowed, and divorced

^b^Others include protestant and catholic

^c^Others include farmer, student

#### Reproductive and reproductive health service-related characteristics

Among the respondents, 407 (74.5%) had one to two live births. Four hundred fourteen (75.8%) of the respondents reported that they had a spontaneous vaginal delivery. About 426 (78%) of the mothers had an intention of having another child in the future. Mothers with a history of a rerun of menses were 300 (54.9%) (Table 2).

**Table 2:** Reproductive and reproductive health service-related history of postpartum mother at Debre Tabor town, South Gondar zone, North West Ethiopia, 2018 (n = 546)

Variables	Category	Frequency	Percent
Parity	1–2	407	74.5
	3–4	122	22.3
	≥ 5	17	3.1
No of a live child	1–2	414	75.8
	3–4	117	21.4
	≥ 5	15	2.7
Do you have an abortion	Yes	51	9.3
	No	495	90.7
Type of abortion (N = 51)	Spontaneous	39	76.5
	Induced	12	23.5
No of abortion (N = 51)	Once	46	90.2
	Two times	5	9.8
Delivery place	Institutional delivery	532	97.4
	Home delivery	14	2.6
Mode of delivery	Spontaneous delivery	414	75.8
	Non-spontaneous delivery	132	24.2
Intention to have another child	Yes	426	78
	No	120	22
Discussed with their husband about FP	Yes	234	42.9
	No	312	57.1
Time interval b/n last and present child (N = 319)	≤ 2 years	51	16
	2.1–3.9 years	69	21.6
	≥ 4 years	199	62.4
Do you have ANC visit for the last pregnancy	Yes	533	97.6
	No	13	2.4
No of ANC visit for last pregnancy (N = 533)	≥ 4 times	460	86.3
	< 4 times	73	13.7
Attend PNC for the last child	Yes	310	56.8
	No	236	43.2
Menses return	Yes	300	54.9
	No	246	45.1
Time of menses return (N = 300)	1–3 months	178	59.3
	4–6 months	76	25.3
	7–9 months	24	8
	10–12 months	22	7.3
Resume sexual intercourse	Yes	499	91.4
	No	47	8.6
Time resume sexual intercourse (N = 499)	1–3 months	445	89.2
	4–6 months	49	9.8
	7–9 months	4	0.8
	10–12 months	1	0.2
Currently, use modern contraceptive	Yes	344	63
	No	202	37
Information about PPFP before delivery	Yes	240	44.0
	No	306	56.0
Information about PPFP after delivery of the last child	Yes	209	38.3
	No	337	61.7
When do you get (N = 209)	Within 24 h of delivery	106	50.7
	3–42 postpartum days	11	5.3
	After 42 PP days	92	44
History of FP	Yes	451	82.6
	No	95	17.4

Among the contraceptive utilizers, injectables were the most commonly used methods accounting for 213 (60.2%) of the total utilization, followed by pills 28%, implants and IUD 9.9% and IUD 1.1% respectively.

Different reasons where mentioned for non-utilization. This includes, unreturning of menses 82 (42.7%) lack of sexual initiation 19.3% the need to have more children 14.6% and fear of contraceptive side effects 12.7%. Majority of the mothers 208 (86.7%) received counseling about PPFP services from ANC clinics, 22.9% from health extension workers and 14.6% from the family planning clinics before the last child was delivered. On the other hand, 56.5% got PPFP counseling from labor and delivery ward 34.9%, from immunization clinic, 25.8% from PNC and 19.1% from health extension worker after the delivery of the last child.

#### Factors associated with postpartum family planning utilization

On bivariate analysis, the factors found to be significantly associated with utilization of PPFP were: age of the mothers, marital status of the mother, educational status of the mother, abortion, a return of menses, intention to have another child and previous history of family planning utilization.

From the variables found to be significant in the bivariate analysis; age of the mothers (25–29) [AOR: 2.004 (95%CI 1.079, 3.722)], marital status (married) [AOR 4.804 (95% CI 1.843, 12.521)], return of menses [AOR: 3.639 (95% CI 2.454, 5.396)] and previous history of family planning utilization [AOR: 2.409 (95% CI 1.474, 3.937)] were found to be significantly associated with utilization of postpartum family planning in multiple logistic regression analysis (Table 3).

**Table 3:** Bivariate and multivariable logistic regression analysis of factors associated with utilization of postpartum family planning at Debra Tabor town, South Gondar zone, North West Ethiopia, 2018 (n = 546)

Variables	Category	PPFP utilization		COR (95% CI)	AOR (95% CI)
		Users	Non-users		
Age	≤ 24	92	38	2.207 (1.192–4.088)*	1.746 (0.88–3.461)*
	25–29	166	80	1.892 (1.086–3.296)	*2.004 (1.079–3.722)*
	30–34	52	53	0.895 (0.482–1.661)	*0.091 (0.456–1.815)*
	≥ 35 (ref)	34	31		
Marital status	Married	337	182	5.29 (2.196–12.747)**	4.804 (1.843–12,521)**
	Others (ref)	7	20		
Educational status	No formal education (ref)	57	56	*	
	Primary education	66	26	2.494 (1.39–4.476)	1.639 (0.842–3.191)
	Secondary education	86	55	1.536 (0.931–2.534)	1.05 (0.595–1.854)
	Tertiary education	135	65	2.040 (1.272–3.274)	1.117 (0.649–1.923)
Previous Abortion	Yes	22	29	0.408 (0.227–0.731)	0.572 (0.3–1.092)
	No (ref)	322	173		
Mode of delivery	Spontaneous (ref)	254	160		
	Non-spontaneous delivery	90	42	1.35 (0.89–2.047)	1.224 (0.769-1.946)
Intention to have another child	Yes	278	148	1.537 (1.019–2.319)*	1.015 (0.615–1.674)
	No (ref)	66	54		
ANC	Yes	339	194	2.796 (0.902–8.665)	2.256 (0.646–7.873)
	No (ref)	5	8		
PNC	Yes	204	106	1.32 (0.93–1.873)	1.138 (0.766–1.692)
	No (ref)	140	96		
Return of menses	Yes	229	71	3.674 (2.549–5.295)**	3.639 (2.454–5.396)**
	No (ref)	115	131		
Previous history of FP	Yes	302	149	2.558 (1.631–4.011)**	2.409 (1.474–3.937)**
	No (ref)	42	53		

Others: single, divorced and widowed

The italic signifies P < 0.05

*P-value < 0.05

**P-value ≤ 0.001

### Discussion

Even though post-partum family planning utilization plays a major role in decreasing unmet need and avoid shortly spaced births, still the utilization is very low.

This study showed that the prevalence of PPFP utilization was 63% (95% CI 59%, 67.4%) which is lower than the studies done at Addis Ababa, Ethiopia 80.3% [11], at Ntchisi District Hospital, Malawi 74.6% [12], the County hospital in rural Kenya 86.3% [13]. This might be due the difference in the socio-demographic characteristics of the respondents, the time gap of the studies and the difference in the study design; in which the last two studies were institutional based studies. It could also be due to the difference in the definition of PPFP utilization in which our study included only modern methods of contraception whereas the first study incorporated traditional as well as modern family planning method use to define utilization status.

On the other hand, the finding was higher than studies that were done in Burundi 20%, Rwanda 51%, Uganda (28%), at Gondar town, Ethiopia (48.4%) and at Kebribeyah Town Ethiopia (12.3%), [14–17]. This might be due to the differences in socio-demographic characteristics and types of data used in which the former two studies used secondary data but the data for our study was a primary. The time gap between the studies might also play a role for the observed differences. Women age 25–29 were more likely to utilize PPFP than their older counter parts. The result is supported by studies conducted at the County hospital in rural Kenya, Uganda, Nigeria and Gondar town Northern Ethiopia [13–15, 18]. This might be due the fact that younger women have more frequent ANC follow up that increased the chance of getting information about PPFP. In addition, older age women may think they could not get pregnant as they are getting older that intern may lower the utilization.

On the contrary, the study conducted in Malawi, showed that there is increased utilization of postpartum family planning as the mother gets older [12]. This difference might be due to the difference in the in culture and socio-economic status of women.

In this study, married mothers were 4.8 times more likely to use postpartum modern contraceptives. This is in line with the study done at Addis Ababa [11]. This might be due to, since married women lived with their husbands, they might start regular sexual intercourse earlier than the non-married couples that may necessitate utilization of PPFP to program the birth of the next child. Mothers whose menses returned were 3.6 times more likely to utilize modern contraceptives. This is in consistency with literatures done in Addis Ababa, Gondar, Northwest Ethiopia, Northern Ethiopia, Gondar town, Gozamen District, East Gojam Zone, Northwest Ethiopia, and Debra Birhan town [11, 15, 19–21]. This could be due to the cultural believe that a return of fertility only occurs along with the return of menses.

Mothers who used modern family planning method before the birth of the indexed child were 2.4 times more likely to utilize PPFP. This is also supported by the study conducted at Addis Ababa, Ethiopia [11]. This could be due to women who have prior family planning utilization has a better understanding of the different types of family planning methods along with the merits and demerits that will enhance their decision making skill towards PPFP utilization. They may also have good attitude towards utilization of family planning.

## Limitations

Utilization status was determined at a point in time which may slightly lower the prevalence.

## Data Availability

The authors declare that the data regarding this manuscript can be accessed as per the request of any interested body and can be submitted for publication in Spring Nature as supplementary materials.

## Abbreviations

ANC: ante natal care
AOR: adjusted odds ratio
CI: confidence interval
COR: crud odds ratio
CPR: contraceptive prevalence rate
DHS: Demographic Health Survey
FP: family planning
LAM: lactation amenorrhea method
MCH: maternal and child health
MDG: millennium development goal
PNC: post natal care
PPFP: post-partum family planning
SPSS: statistical package for social scientists
WHO: World Health Organization
